# Human Endogenous Retroviruses Are Preferentially Expressed in Mononuclear Cells From Cord Blood Than From Maternal Blood and in the Fetal Part of Placenta

**DOI:** 10.3389/fped.2020.00244

**Published:** 2020-05-14

**Authors:** Massimiliano Bergallo, Luca Marozio, Giovanni Botta, Annalisa Tancredi, Valentina Daprà, Ilaria Galliano, Paola Montanari, Alessandra Coscia, Chiara Benedetto, Pier Angelo Tovo

**Affiliations:** ^1^Pediatric Laboratory, Department of Pediatric Sciences and Public Health, University of Turin, Turin, Italy; ^2^Department of Pediatric Sciences and Public Health, University of Turin, Turin, Italy; ^3^Department of Surgical Sciences, Obstetrics and Gynecology 1, University of Turin, Turin, Italy; ^4^Department of Pathology AOU Città della Salute e della Scienza di Torino, Turin, Italy; ^5^Neonatology Unit, Department of Pediatric Sciences and Public Health, University of Turin, Turin, Italy

**Keywords:** HERV human endogenous retroviruses, PCR, placenta, cord blood, mRNA expression

## Abstract

**Background:** Placenta shows high transcription levels of human endogenous retroviruses (HERVs) that are overexpressed during embryonic and fetal development.

**Methods:** In order to gather further information on the degree of HERV activation in maternal and fetal tissues we assessed the transcription levels of pol genes of HERV-H, -K, and -W in PBMCs of newborns and their mothers as well as in chorion (fetal part) and decidua basalis (maternal part) of the placenta using a real time PCR assay.

**Results:** Transcripts of pol genes of the three HERV families were significantly higher in mononuclear cells from cord blood than from maternal blood and in the fetal part than in the maternal part of the placenta.

**Conclusions:** The HERV over-expressions in cells and tissues of the offspring are further clues that they play pivotal physiologic roles during early life events and suggest that HERV-driven abnormalities of pregnancy and fetal development may derive mostly from the conceptus, not from the mother.

## Introduction

Human endogenous retroviruses (HERVs) derive from ancestral infections of somatic cells with subsequent integration into germ line of primates more than 25 millions years ago. During evolution, mutations, recombinations, insertions and/or deletions have rendered their sequences unable to produce infectious virions. However, HERVs maintain their retroviral structure with gag, pol, and env genes, flanked by two non-coding long terminal repeats, that function as control regions (1). Most HERV sequences are inactive, but some encode proteins of viral origin. HERV proviruses can modulate the expression of adjacent cellular genes and their transcripts, through viral reverse transcriptase and integrase, may generate novel insertions into the DNA (2).

HERVs represent 8% of our genome, but their functions remain poorly defined. They have been associated with a large array of diseases, such as cancers, autoimmune diseases and neurologic disorders. On the other hand, their massive and prolonged persistence within our DNA suggests that they also have some beneficial roles. For instance, accumulating evidence highlights the importance of HERV elements in placentation (3–6), in the early phases of embryogenesis (7), in fetal development and in maintaining stemness of pluripotent cells (8).

The human placenta is composed of various cell types, that include extravillous and villous cytotrophoblasts. The latter can further be differentiated into an overlaying structure, referred to as the syncytiotrophoblast. This regulates the proper maternal-fetal exchange of nutrients and the production of important hormones, such as human chorionic gonadotropin and human placental lactogen whose production is regulated by retroviral elements (9). The placenta actively expresses a number of HERV genes. Among these, two envelope proteins, termed syncytin-1 (3) and syncytin-2 (4), are involved in normal placenta formation, drive the fusion between villous cytotrophoblast and the syncytiotrophoblast layer (5, 6), and may influence the feto-maternal tolerance (10). Genes similar to syncytins and with comparable functional properties are expressed in the placenta of various eutherian mammals. This suggests a convergent evolutionary process and represents a typical example how the host has co-opted retroviral elements to his own survival (11).

Several retroviral groups have been identified (2). Among these HERV-H, HERV-K, and HERV-W are those most widely studied. We previously observed that pol genes of these three retroviral groups are overexpressed in peripheral blood mononuclear cells (PBMCs) from cord blood, particularly in preterm newborns in inversed correlation with gestational age (12). The aims of the current project were to assess whether this enhanced HERV activation is present at birth also in the mother. Furthermore, since the activation of HERV-pol genes in the placenta has not been investigated and the localization of their transcripts in different placental cell populations is unkown, we evaluated the expression of pol genes of HERV-H, -K, and -W in both the maternal and fetal components of placenta.

## Materials and Methods

### Peripheral Blood Mononuclear Cells

PBMCs were collected after an uneventful, at term pregnancy and vaginal delivery from parturients and from cord blood of newborns without any clinical or laboratory abnormalities.

### Placental Tissues

Placental specimens were obtained from uneventful, at term (>37 weeks of gestation) pregnancies after vaginal or Cesarean delivery. Placenta tissues were washed with Hank's solution to remove contaminating blood. The decidua basalis (i.e., the maternal part of the placenta) and the chorion (i.e., the fetal part of the placenta, represented mainly by syncytiotrophoblast) were macroscopically identified and separated by an expert pathologist (GB) and confirmed by a microscopic examination. Small samples (~5–10 mg wet weight) of each part of the placental tissue were placed in dry tubes and immediately processed for RNA extraction.

### Total RNA Extraction

Total RNA was extracted from chorion, decidua basalis, and PBMCs using the automated extractor Maxwell (Promega, Madison, WI) following the RNA Blood Kit protocol without modification. This kit provides treatment with DNase during the RNA extraction process. Four hundred nanograms of total RNA was reverse-transcribed with 2 μl of buffer 10X, 4.8 μl of MgCl_2_ 25 mM, 2 μl ImpromII (Promega), 1 μl of RNase inhibitor 20U/l, 0.4 μl random hexamers 250 μM (Promega), 2 μl mix dNTPs 100 mM (Promega), and dd-water in a final volume of 20 μl. The reaction mix was carried out in a GeneAmp PCR system 9,700 Thermal Cycle (Applied Biosystems, Foster City, CA, USA) under the following conditions: 5 min at 25°C, 60 min at 42°C and 15 min at 70°C for the inactivation of enzyme; the cDNAs were stored at −80° until use. For control of genomic DNA contamination we amplified directly RNA extracts without reverse transcription.

### Transcription Levels of Pol Genes of HERV-H, -K, and -W

Relative quantification of mRNA expression of HERV-H, -K, and -W was achieved by means of PCR real time Taqman amplification and normalization to glyceraldehyde-3-phosphate dehydrogenase (GAPDH) using the ABI PRISM 7,500 real time system (Life technologies, Texas, USA). GAPDH was chosen as reference gene being the most stable among 9 reference genes (13) and already used in our previous studies (12, 14, 15). Forty nanograms of cDNA were amplified in a 20 μl total volume reaction containing Go-Taq mastermix probe (Promega), 500 nmol of specific primers and 200 nmol of specific probes. The following primers and probes for pol genes were used: HERV-K primers (KPOLF-5′-CCACTGTAGAGCCTCCTAAACCC-3′) (KPOLR-5′-TTGGTAGCGGCCACTGATTT-3′) and probe (KPOLP-6FAM-CCCACACCGGTTTTTCTGTTTTCCAAGTTAA-TAMRA) as reported by Schaban et al. (16); for HERV-H primers (HPOLF-5′- TGGACTGTGCTGCCGCAA-3′) (HPOLR-5′-GAAGSTCATCAATATATTGAATAAGGTGAGA-3′) and probe (HPOLP-6FAM- TTCAGGGACAGCCCTCGTTACTTCAGCCAAGCTC-TAMRA; HERV-W primers (WPOLF-5′-ACMTGGAYKRTYTTRCCCCAA-3′) (WPOLR-5′- GTAAATCATCCACMTAYYGAAGGAYMA-3′) and probe (WPOLP-6FAM-TYAGGGATAGCCCYCATCTRTTTGGYCAGGCA-TAMRA); GAPDH primers (GAPDHF-5′-CCAAGGTCATCCATGACAAC-3′) (GAPDHR-5′- GTGGCAGTGATGGCATGGAC-3′) and probe (GAPDH-6FAM- TGGTATCGTGGAAGGA-3′ MGB). The established assays use probes and primers designed by Primer Express TM software version 3.0 (Applied Biosystems, Foster City, USA). Basic Local Alignment Search Tool (BLAST) analysis confirmed no cross-reaction between HERV primers.

The amplifications were run in a 96-well plate at 95°C for 10 min, followed by 40 cycles at 95°C for 15 s and at 60°C for 1 min. Furthermore, in order to confirm that there was no DNA genomic contamination, control PCR was performed with RNA before reverse transcription using the same primers and probes described above. Each sample was run in triplicate. Relative quantification of target gene expression was performed with the ΔCt method. Using 40 ng of cDNA in amplification we obtained Ct value from 26 to 30.4. These Ct values correspond to a good performance of real time PCR. Since we measured Ct for every target in all the samples tested, we argued that our methods were suitable for HERV detection and quantification. Results were expressed as 1/ΔCt.

### Statistical Analysis

Mann-Whitney test was used to compare the transcriptional levels of pol genes of every HERV group in chorion vs. decidua basalis as well as in PBMCs from cord blood vs. maternal blood. Statistical analyses were done using the Prism software (GraphPad Software, La Jolla, CA). In all analyses, *p* < 0.05 was taken to be statistically significant.

## Results

PBMCs were collected after vaginal delivery from 35 women and from cord blood of 47 newborns (13 males, 34 females). The gestational age was comparable between the maternal and the neonatal population (median gestational age 281 days, range 270–292 vs. 277 days, range 260–295, respectively). Placental tissue was collected from 57 women at delivery (24 vaginal deliveries, 33 Cesarean sections).

The pol genes of HERV-H, HERV-K, and HERV-W were always transcriptionally active in PBMCs from mothers and newborns as well as in the chorion and in the decidua basalis.

**Figure 1:**
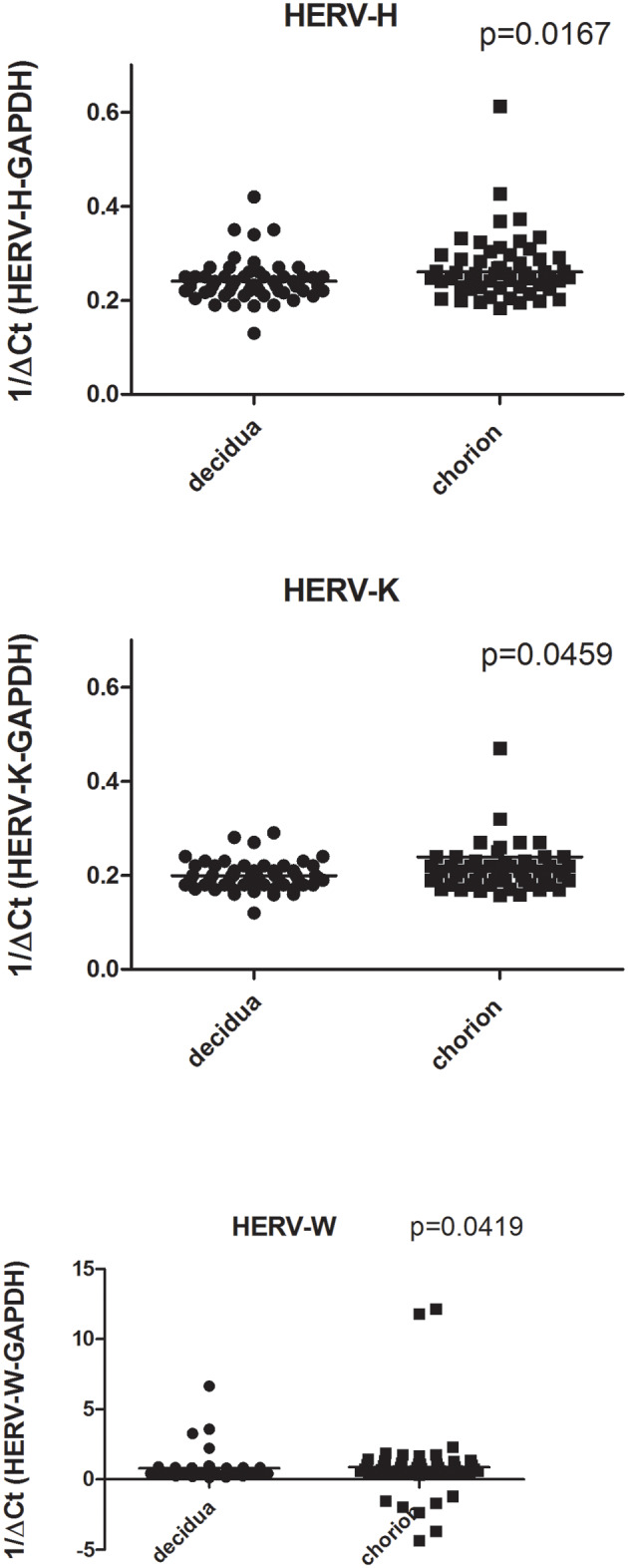
Transcription levels of pol genes of HERV-H, HERV-K, and HERV-W in decidua basalis and chorion from placentae of uneventful, at term pregnancies. Data are represented as black dot. Relative pol levels were assessed by real-time PCR and represented by 1/ΔCt. Statistical significance was calculated by Mann-Whitney test.

As detailed in Figure 1 the number of transcripts of pol genes of HERV-H, HERV-K, and HERV-W were significantly higher in chorion than in decidua basalis. In particular, the HERV-H-pol values (mean +/– SD) were: 0.26 +/– 0.14 in chorion vs. 0.24 +/– 0.04 in decidua basalis, *p* = 0.0167; the HERV-K-pol values were: 0.23 +/– 0.19 in chorion vs. 0.19 +/– 0.02 in decidua basalis, *p* = 0.0459; and the HERV-W-pol values were: 0.87 +/– 2.55 in chorion vs. 0.79 +/– 1.03 in decidua, *p* = 0.0419, with the largest range of values (including a few negative findings). The preferential HERV expression in the fetal part of placenta was not influenced by mode of delivery (data not shown).

**Figure 2:**
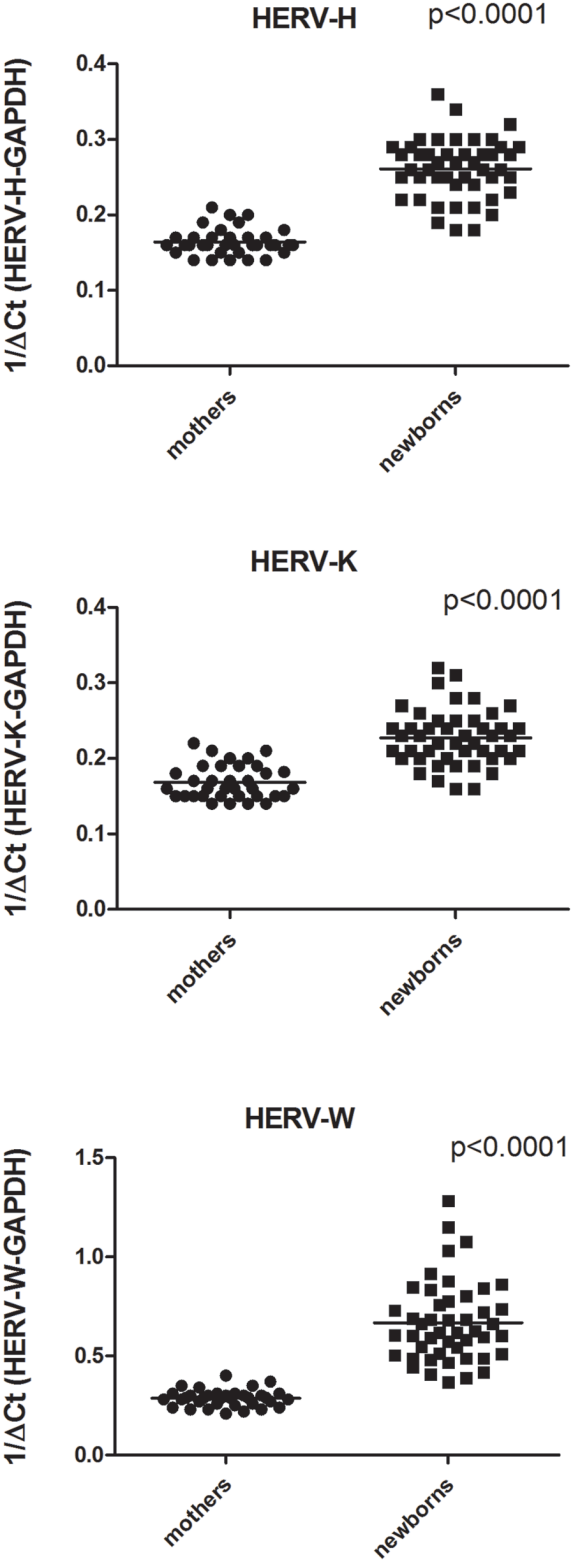
Transcription levels of pol genes of HERV-H, HERV-K, and HERV-W in mononuclear cells from peripheral blood of parturients and from cord blood of newborns collected after an at term pregnancy and vaginal delivery. Data are represented as black dot. Relative pol levels were assessed by real-time PCR and represented.

As detailed in Figure 2 the expression levels of pol genes of HERV-H, HERV-K, and HERV-W were significantly higher in PBMCs from newborns than from parturients. In particular, the HERV-H-pol values (mean +/– SD) were: 0.26 +/– 0.03 in newborns vs. 0.16 +/– 0.02 in parturients, *p* < 0.0001; the HERV-K-pol values were: 0.22 +/– 0.04 in newborns vs. 0.17 +/– 0.02 in parturients, *p* < 0.0001; and the HERV-W-pol values were: 0.66 +/– 0.2 in newborns vs. 0.28 +/– 0.04 in parturients, *p* < 0.0001. No significant differences were found in newborns between males and females (data not shown).

## Discussion

The results of this study document a significantly higher expression of pol genes of all the three HERV groups in the chorion (the fetal part of the placenta) than in the decidua basalis (maternal part). Our findings on HERV-pol genes are consistent with other studies, focused on HERV-env genes, demonstrating that their transcripts are mainly localized in villous cytotrophoblast and/or syncytiotrophoblast (5, 6, 17, 18). Notably, the expression of endogenous retroviruses occurs mostly in the fetal part of the placenta not only in invasive placental types with syncytiotrophoblast formation, such as the hemochorial placenta of human beings, but also in epitheliochorial placentas, such as in horses (19), where the syncytiotrophoblast is absent and fetal trophoblast cells are simply apposed to the intact maternal epithelium. This suggests that the preferential fetal expression of HERVs is an important feature for the placental structure and functions that has been preserved over the phylogenesis.

Present results for the first time also show that at birth the transcriptional levels of pol genes of HERV-H, HERV-K, and HERV-W are significantly higher in PBMCs from cord blood than from maternal blood. Therefore, our findings evidence that during pregnancy there is an enhanced HERV transcription that typically involves cells and tissues of fetal origin, whereas cells and tissues of maternal origin are less interested by the phenomenon. Several findings support this consideration. HERV-K is efficiently transcribed during human embryogenesis beginning at the 8-cell stage and continuing until the blastocyst outgrowths (3). Besides the aforementioned syncytins, recently a new tamed retroviral envelope protein produced from the fetus and then shed extracellularly into the maternal blood has been identified (8). We found that HERV-pol transcripts are significantly higher in PBMCs from premature than in full-term neonates in inversed correlation with gestational age, while postnatally they decrease with no further variations throughout the first decade of life (12). Also in rabbits the placenta-specific expression of endogenous retroviruses shows a 4-fold decrease with the progression of gestational age (20). It must be underlined that HERVs are efficiently transcribed in germ cells and pluripotent stem cells (2, 8) where they contribute to maintain their pluripotency circuit and cell fate reprogramming (21). Cord blood is reach of CD34+ hematopoietic and progenitor stem cells particularly in preterm newborns (22). The high proportion of stem cells in cord blood might thus explain, at least in part, why HERV transcription is higher in neonatal than in maternal PBMCs.

Taken together all these findings suggest that HERVs play crucial physiologic roles in the first life events, starting from the preimplanted blastocyst and continuing from placentation till birth through the placental tissues and the fetal development and differentiation. Indeed the primers and probes we used encompass several HERV-pol sequences, with the consequent limit that their localization in specific loci could not be identified. PBMCs from mothers and newborns and placental tissues were obtained from partly different populations. Furthermore, we did not assess their protein-coding capacity, though this does not undermine their potential importance, since also in placenta HERVs can act as non-coding regulatory elements able to promote or enhance the activity of neighboring cellular genes (10). In general, the emerging contribution of HERVs to a variety of ontogenetic processes may explain why a substantial portion of the genome is occupied, from millions of years, by retroviruses.

The underlying mechanisms responsible for the increased HERV transcription in the placenta and during the intrauterine life remain to be fully elucidated. HERV expression is regulated by epigenetic mechanisms, such as the methylation processes of DNA (23). The placental tissues are characterized by DNA hypometylation that triggers HERV transcription (24) and decreases with the progression of pregnancy (25). In contrast, pathologic placentae are characterized by epigenetic hypermethylation of HERV promoters (26). Notably, both the DNA hypomethylation and the HERV overexpression are inversely correlated with gestational age (12, 27). Furthermore, sexual hormones contribute to the regulation of HERVs (28), therefore, the peculiar hormonal patterns occurring throughout pregnancy might represent typical temporary factors conditioning HERV activation.

The present high placental and fetal HERV transcripts were detected in physiological conditions. Alterations of HERV activity might however result in failures or abnormalities of pregnancy or in disturbaces of offspring development. For instance, pre-eclampsia (PE) is a typical pregnancy disorder associated with a defect in placentation that causes important maternal and neonatal morbidity and mortality. Syncytin-1 and −2 transcripts are reduced in placental tissues of women with PE (29, 30). Dysregulations of HERV gene levels were found in placentae of patients with intrauterine growth restriction (31). Furthermore, maternal smoking, typically associated with impaired fetal development, has a significant negative impact on the physiologic overexpression of HERVs in newborns (15).

In general, the preferential HERV expression in cells and tissues of the offspring rather than of the mother suggests that, in a number of instances, abnormalities of pregnancy and of fetal development may result from HERV-driven alterations deriving mostly from the conceptus, even if targeted by maternal factors, such as environmental and hormonal factors. For instance, we looked at possible abnormal HERV activation in blood samples from women with recurrent miscarriages, but we did not find any alterations (unpublished data). In contrast, enhanced HERV expression is associated with development and invasion of several cancers, e.g., the up-regulation of syncytin-1 promotes the malignant transformation of hydatidiform moles (32) and the spread of endometrial carcinoma (33), and all malignancies of placental origin (including those of late appearance) derive from cells of the conceptus, not of the mother.

## Data Availability Statement

The raw data supporting the conclusions of this article will be made available by the authors, without undue reservation, to any qualified researcher.

## Ethics Statement

The subjects included were asked to sign an informed consent. The study was approved by the local Ethical Committee (Comitato Etico Interaziendale AOU Città della Salute e della Scienza di Torino—AO Ordine Mauriziano di Torino—ASL Città di Torino. Protocol no. 0067257–2018). This study was carried out in accordance with the recommendations of the Declaration of Helsinki, and all parents gave informed written consent. The protocol was approved.

## Author Contributions

MB, LM, and PT contributed in conceptualizing the study design. MB and PT prepared the first draft of the manuscript. LM, AT, CB, and AC enrolled the study populations, obtained their informed consent, were responsible for ethical approval, and collected placentae and PBMCs. GB separated placental tissues. MB, VD, IG, and PM were involved in processing samples, data collection, and data analysis. All authors approved the final manuscript.

## Conflict of Interest

The authors declare that the research was conducted in the absence of any commercial or financial relationships that could be construed as a potential conflict of interest.
